# Supercapatteries as Hybrid Electrochemical Energy Storage Devices: Current Status and Future Prospects

**DOI:** 10.3390/molecules29010243

**Published:** 2024-01-02

**Authors:** Subarna Rudra, Hyun Woo Seo, Subrata Sarker, Dong Min Kim

**Affiliations:** Department of Materials Science and Engineering, Hongik University, Sejong 30016, Republic of Korea; subarna@mail.hongik.ac.kr (S.R.); godson@mail.hongik.ac.kr (H.W.S.)

**Keywords:** electrochemical energy storage, supercapacitors, batteries, hybrid storage, supercapatteries

## Abstract

Among electrochemical energy storage (EES) technologies, rechargeable batteries (RBs) and supercapacitors (SCs) are the two most desired candidates for powering a range of electrical and electronic devices. The RB operates on Faradaic processes, whereas the underlying mechanisms of SCs vary, as non-Faradaic in electrical double-layer capacitors (EDLCs), Faradaic at the surface of the electrodes in pseudo-capacitors (PCs), and a combination of both non-Faradaic and Faradaic in hybrid supercapacitors (HSCs). EDLCs offer high power density but low energy density. HSCs take advantage of the Faradaic process without compromising their capacitive nature. Unlike batteries, supercapacitors provide high power density and numerous charge–discharge cycles; however, their energy density lags that of batteries. Supercapatteries, a generic term that refers to hybrid EES devices that combine the merits of EDLCs and RBs, have emerged, bridging the gap between SCs and RBs. There are numerous articles and reviews on EES, and many of those articles have emphasized various aspects of HSCs and supercapatteries. However, there are no recent reviews that dealt with supercapatteries in general. Here, we review recently published critically selected articles on supercapatteries. The review discusses different EES devices and how supercapatteries are different from others. Also discussed are properties, design strategies, and future perspectives on supercapatteries.

## 1. Introduction

Our earth’s climate is changing, primarily due to anthropogenic global warming due to the burning of fossil fuels. It will continue to change at that pace or even more quickly until we take corrective measures [1]. Fortunately, we have started acknowledging the negative impacts of climate change on our environment [2]. As such, the major world powers and the scientific community have focused on renewable energy to curb fossil fuels, a primary culprit in climate change. Since renewable energies are intermittent energy sources, they require energy storage devices to maintain a steady supply. Recently, electric vehicles (EVs) have become increasingly popular, as they are not an active source of carbon emissions and depend on an electric grid or solar array at an invariable or reduced cost. As a result, there has been a great interest in developing efficient electrochemical energy storage (EES) devices.

**Figure 1:**
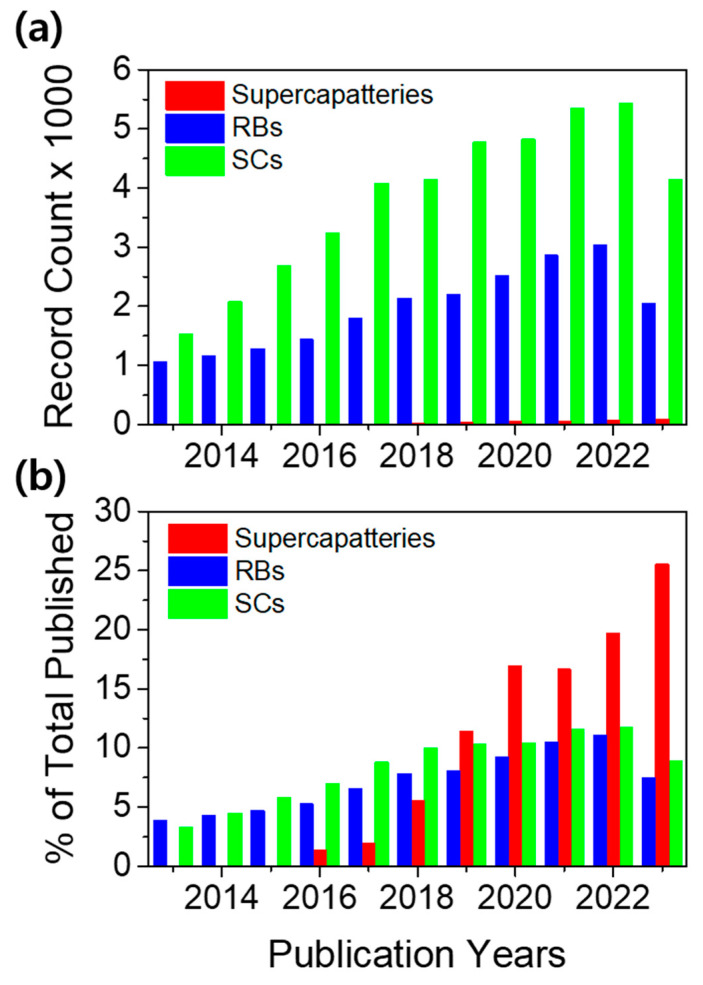
(**a**) Number of articles and (**b**) percent of the total number of articles published on supercapatteries, rechargeable batteries (RBs), and supercapacitors (SCs) over the last ten years (from 2013 to 2023). The data were collected by searching for articles using the keyword “supercapatteries” in the Web of Science Core Collection (Document Search—Web of Science Core Collection).

**Figure 2:**
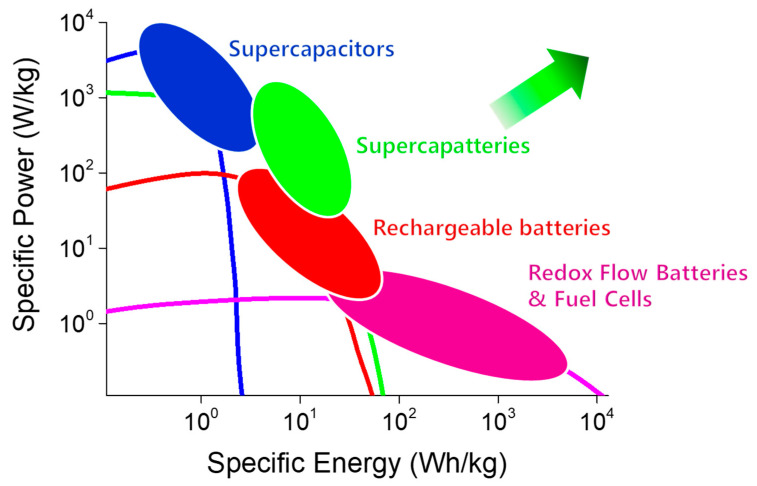
Ragone plot of different energy storage devices showing relative energy and power densities for supercapacitors, rechargeable batteries, redox flow batteries, fuel cells, and supercapatteries [16].

Among EES technologies, rechargeable batteries (RBs) and supercapacitors (SCs) are the two most desired candidates for powering a range of electrical and electronic devices [3,4,5,6,7,8,9,10]. RBs operate on Faradaic processes, whereas the underlying mechanism of SCs varies, as non-Faradaic in electrical double-layer capacitors (EDLCs), Faradaic at the surface of the electrodes in pseudo-capacitors (PCs), and a combination of both non-Faradaic and Faradaic in hybrid SCs (HSCs) [3,11]. EDLCs offer high power density but low energy density. HSCs take advantage of the Faradaic process without compromising their capacitive nature. Unlike batteries, supercapacitors provide high power density and numerous charge–discharge cycles; however, they lag batteries in energy density. To take advantage of the merits of both RBs and SCs, researchers have focused on merging the two technologies into a single device known as a “supercapattery” (= supercapacitor + battery) [12,13,14,15,16], a generic term used to identify a unique category of energy storage devices that offer high energy density like an RB without compromising the ability to deliver the high power density and large cyclability of EDLCs. Though supercapatteries are relatively new compared to RBs and SCs, supercapatteries have received significant attention, as evidenced by the exponential growth in related publications over the past ten years (Figure 1). The advantage of supercapatteries over other EES technologies is evident in the Ragone plots in Figure 2, which compare the power and energy densities of several typical EES systems. However, it is often difficult to distinguish between supercapatteries and other hybrid EES devices due to their overlapping properties.

Here, we review selected articles on supercapatteries encompassing the characteristics of RBs and SCs with high energy and power densities, respectively. The review discusses different types of electrochemical energy storage devices in terms of mechanisms and materials to form a supercapattery. The properties of and design strategies for supercapatteries, along with their electrochemical characterization, are also discussed. The final section summarizes the review with a perspective on supercapatteries in the future.

## 2. Some Important Definitions and Parameters of EES

### 2.1. Capacitance and Charge of an Electrode

The capacitance (*C*) of a dielectric capacitor depends on the area (*A*) of the conducting electrodes and the distance (*d*) between the electrodes [11]:

(1) $$C=\frac{\varepsilon_{r}\varepsilon_{0}A}{d}$$

where $\varepsilon_{r}$ and $\varepsilon_{0}$ are the relative and vacuum permittivity, respectively. According to Equation (1), the capacitance of EDLCs increases as *A* increases and *d* decreases. The behavior of an electrode/electrolyte interface is analogous to that of a capacitor. At a voltage (*V*) across a capacitor with a capacitance of *C*, the total amount of charge (*Q*) stored is [17]

(2) Q=CV

When two capacitors with capacitance *C*_1_ and *C*_2_ are connected in series, their total capacitance (*C*_T_) is expressed as

(3) $$\frac{1}{C_{T}}=\frac{1}{C_{1}}+\frac{1}{C_{2}}$$

If *C*_1_ and *C*_2_ are the same, that is, $C_{1}=C_{2}=C$, then Equation (3) becomes

(4) $$C_{T}=\frac{C}{2}$$

However, if the two capacitors are different and *C*_1_ is much smaller than *C*_2_ ($C_{1}\ll C_{2}$), then $C_{1}+C_{2}≅C_{2}$ and Equation (3) becomes

(5) $$C_{T}≅C_{1}$$

That is, the total capacitance of a series combination of two capacitors with different capacitances will be dominated by the capacitor with a smaller capacitance.

Moreover, differentiating Equation (2) with respect to time (*t*) gives Equation (7).

(6) $$\frac{dQ}{dt}=C\frac{dV}{dt}+V\frac{dC}{dt}$$

Since i=dQ/dt, and considering that *C* does not change with time, Equation (7) can be written as [18]:

(7) $$C=\frac{dQ}{dt}/\frac{dV}{dt}=i/\frac{dV}{dt},$$

which can be written for ν=dV/dt as

(8) $$C=\frac{i}{\nu }$$

### 2.2. Galvanostatic Charge–Discharge (GCD)

**Figure 3:**
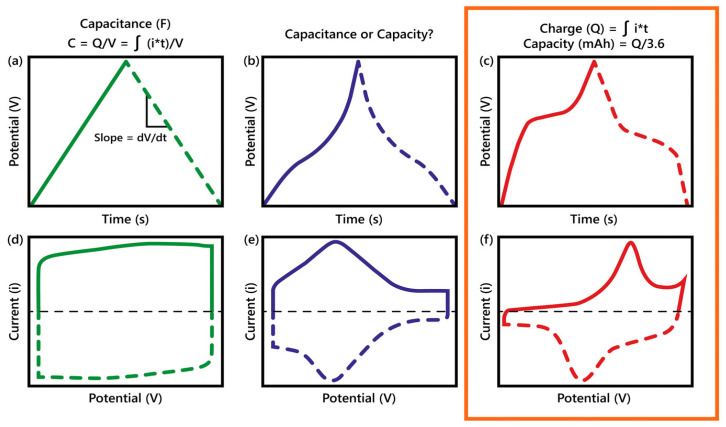
Archetypal electrical output behavior of the three main types of electrodes, including (**a**,**d**) electrical double-layer, (**b**,**e**) pseudocapacitive, and (**c**,**f**) battery types. (**a**–**c**) Schematic of galvanostatic charge–discharge (GCD) profiles showing linear and nonlinear responses with time and (**d**–**f**) corresponding cyclic voltammetry (CV) profiles [21].

In the Galvanic technique, voltage response is measured by applying a constant current within a potential window bounded by initial and final voltages. Galvanostatic charge–discharge (GCD) is one of the most effective techniques to evaluate the capacity and capacitance of RBs and SCs at a constant current. According to Equation (7), during the charging process of a capacitor at a constant current, the voltage increases at a constant rate. Conversely, during the discharging process, the voltage decreases at a constant rate. This results in a triangular curve in GCD, as depicted in Figure 3a [19]. Thus, one can calculate the capacitance of an electrode material from the discharge current (i) and the slope (dV/dt) of the discharging part of the triangular GCD curve [20].

### 2.3. Cyclic Voltammetry (CV)

Cyclic voltammetry (CV) is one of the most popular electrochemical techniques for investigating new electrochemical systems or materials. In CV measurement, a linear potential sweep or ramp is applied where the potential (*V,* V) is changed from an initial value *V*_i_ (V) [22]:

(9) $$V=V_{i}+\nu t$$

Therefore, the current flowing through a capacitor is in a linear relationship with ν but independent of *V*. For a constant *C*, Equation (8) gives the rectangular *i-V* plots as shown in Figure 3d. However, CV plots for pseudocapacitive and faradaic electrodes are not linear, as shown in Figure 3e,f. Analysis of the CV profile or voltammogram can provide much useful information, including the cyclability of the process, the total capacitance, the optimum potential window, the electrochemical kinetics of electrodes, and an ability to distinguish the capacitive and diffusion-limited charge storage mechanisms by altering the sweep rate [19]. The capacitance of the electrodes under study can be estimated by integrating the CV curves according to Equation (10) [20]:

(10) $$c=\frac{\int_{V_{1}}^{V_{2}}idV}{\nu \Delta V},$$

where i is the discharge current, i.e., the current below the *x* axis, ν is the scan rate, and $\Delta V=V_{2}-V_{1}$ is the operating discharge potential range.

### 2.4. Mass and Charge Balance

The stored charge of an electrode can be expressed by the following equation:

(11) $$Q=C_{s}\;m\;∆V,$$

where m, $C_{s}$, and ∆V represent the mass, specific capacitance, and potential window obtained from the charging/discharging process in a three-electrode configuration. Equation (11) can be written for positive and negative electrodes as

(12) $$Q_{+}=C_{s_{+}}\;m_{+}\;∆V_{+},$$

and

(13) $$Q_{-}=C_{s_{-}}\;m_{-}\;∆V_{-},$$

respectively. According to Equations (3)–(5), the overall capacitance of a supercapacitor is maximized when both electrodes have similar capacitance. This can be achieved by ‘mass balancing’ with data obtained from half-cell experiments. In this case, it is critical to optimize the cell voltage to conserve charges using the relationship $Q_{+}=Q_{-}$. Thus, from Equations (12) and (13), it can be shown that

(14) $$\frac{m_{+}}{m_{-}}=\frac{C_{s_{-}}{∆V}_{-}}{C_{s_{+}}{∆V}_{+}}$$

Equation (14) can be used to balance the charge of the electrodes in asymmetric SCs and even supercapatteries to maximize the capacitance of the device [23].

### 2.5. Energy Density

**Figure 4:**
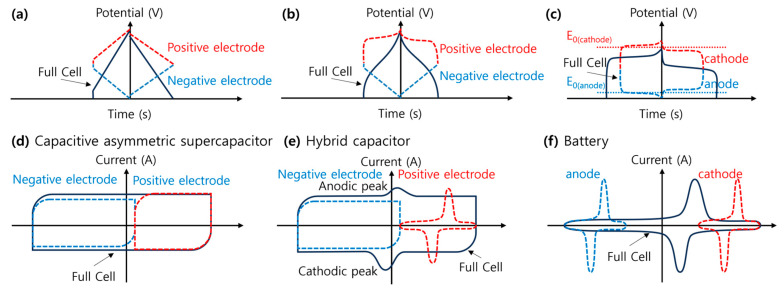
Schematic illustration of the typical GCD (up) and CV (bottom) curves depicting characteristics of (**a**,**d**) a capacitive asymmetric supercapacitor, (**b**,**e**) a hybrid capacitor, and (**c**,**f**) a battery [24].

The electric energy stored in SCs, i.e., the energy density (*E*, Wh/kg), can be evaluated by integrating the GCD curves. For EDLCs and PCs with linear GCD curves, the integration turns into the calculation of triangle areas, as shown in Figure 4d; therefore, the energy density can be calculated by [20].

(15) $$E=\int QdV=\int_{0}^{V}CVdV=\frac{1}{2}CV^{2}$$

However, in the case of HSC with a nonlinear GCD curve, as shown in Figure 4e, the energy density cannot be calculated simply by using Equation (15) due to the nonlinear change in V. In this case, energy density should be calculated following Equation (16) [20].

(16) $$E=\int QdV=\int_{t_{1}}^{t_{2}}iV_{t}dt=i\int_{t_{1}}^{t_{2}}V_{t}dt$$

Equation (16) considers all discharge times (t) in hours (h) and discharge voltages ($V_{t}$) for the calculation after the initial IR drop. In Equation (16), $t_{1}$ is the time after the initial IR drop, $t_{2}$ is the time when the discharge is finished, and i is the constant current applied to the supercapacitor.

### 2.6. Power Density

The power density (*P*, W/kg) values of SCs can be calculated according to Equation (17) [23],

(17) $$P=\frac{E}{t},$$

where t is the discharge time in hours (h).

### 2.7. Ragone Plots

The performance of EES devices is compared using Ragone plots, which show both power density (time required to charge and discharge) and energy density (storage capacity), as shown in Figure 2. However, the plots do not show cyclability, a critical metric of EES devices [25,26].

### 2.8. Capacitive and Diffusive Storage

**Figure 5:**
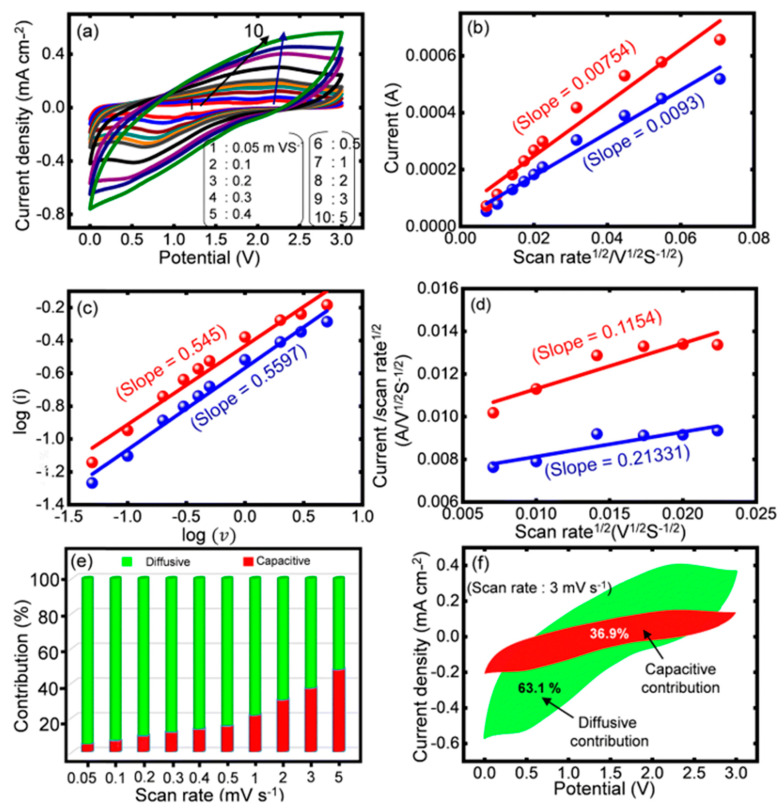
(**a**) CV profiles at different scan rates from 1 to 10 mV/s. (**b**) Peak current vs. square root of the scan rate (n). (**c**) Plot of log(i) vs. log(n). (**d**) Dunn’s plots. (**e**) Deconvoluted percentage contributions of capacitive and diffusive currents at different scan rates. (**f**) Representative CV profile at a scan rate of 7 mV/s marked with the capacitive and diffusive contributions recorded for the NiCo_2_S_4_-NiCo_2_O_4_ electrode in 6 M KOH [23].

To understand the capacitive and diffusive contributions to the total discharge capacity of electrode material, CV curves can be recorded at different low scan rates in a certain potential window, as shown in Figure 5a. The presence of current peaks is due to the conversion/reconversion reactions at the electrode. The anodic and the cathodic peak currents (Figure 5b) increase with increasing scan rate, according to the power law given below [23]:

(18) $$i=a\nu^{b},$$

where *i* is the current response at a particular peak and *a* and *b* are constants. Equation (18) can be linearized as follows:

(19) $$log\left(i\right)=log\left(a\right)+b\;log(\nu )$$

The value of *b* indicates whether the current is diffusive, that is, diffusion-controlled (*b* = 0.5), or capacitive, that is, surface-controlled (*b* = 1) [27]. However, the value of b lies between 0.5 and 1.0 for mixed processes (Figure 5c). According to Dunn’s equation, the peak current and the scan rates are related, as shown below [23,27]:

(20) $$i=k_{1}{\nu +k_{2}\nu }^{0.5},$$

where $k_{1}$ and $k_{2}$ are constants. Equation (20) can be rearranged into Equation (21) to estimate the values of $k_{1}$ and $k_{2}$ from the plot of iν0.5 against $\nu^{0.5}$ as shown in Figure 5d.

(21) iν0.5=k1ν0.5+k2

Once $k_{1}$ and $k_{2}$ are known, the percentage of capacitive and diffusive current can be estimated (Figure 5e) and the CV profile can be deconvoluted for both contributions (Figure 5f).

### 2.9. Electrochemical Impedance Spectroscopy (EIS)

Electrochemical impedance spectroscopy (EIS) is a powerful method for characterizing the electrical properties of materials and their interfaces. In EIS, a small-amplitude modulated voltage, V(ω, t), is applied over a wide range of frequencies (f=ω/2π) and the corresponding current, i(ω, t), is recorded, or vice versa. The resultant impedance Z(ω) of the system is calculated following [26]:

(22) $$Z(\omega )=\frac{V(\omega ,t)}{i(\omega ,t)}$$

The impedance is often represented by the real part $Z^{\prime }$ and the imaginary part $Z^{\prime \prime }$ as a complex number.

(23) $$Z=Z^{\prime }+{jZ}^{\prime \prime }$$

A detailed account of electrochemical impedance and complex capacitance to interpret electrochemical capacitors was given by Itagaki et al. [28]. Like complex impedance, complex capacitance can also be expressed as

(24) $$C=C^{\prime }+{jC}^{\prime \prime }$$

The relationship between Equation (23) and Equation (24) is defined by C=1/jωZ, where $C^{\prime }=-Z^{\prime \prime }/\omega (Z^{\prime 2}+Z^{\prime \prime 2})$ and $C^{\prime \prime }=-Z^{\prime }/\omega (Z^{\prime 2}+Z^{\prime \prime 2})$.

**Figure 6:**
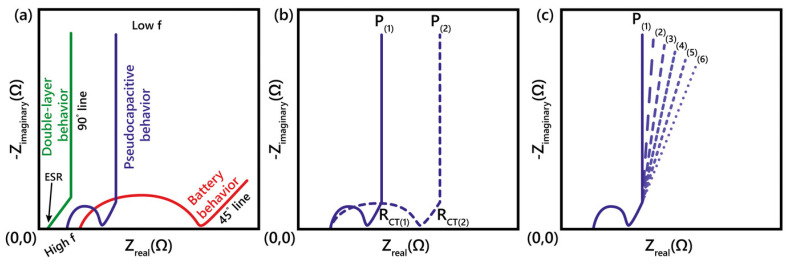
(**a**) Typical Nyquist plot representations for an EDLC (green curve), pseudocapacitive material (blue), and a battery (red). ESR, 45°, and 90° lines are marked. (**b**) Example spectra confirming real charge transfer resistance (RCT) with EIS at two different potentials (solid and dotted blue curves), in contrast to (**c**) where interfacial impedance would lead to constant RCT at all potentials. Pseudocapacitive materials will show minimal diffusion-limited behavior (meaning no diffusion limitations relative to batteries) [21].

Figure 6 shows typical EIS spectra in the complex plane distinguishing different charge storage mechanisms [21].

### 2.10. Notation for Electrodes of EES

Several terms have often been used interchangeably in EES to denote electrodes, like positrode for the positive electrode and negatrode for the negative electrode, since G. Z. Chen proposed these terms in 2015 to avoid any confusion among newcomers to the EES community [18]. It is important to note that the terms cathode or anode are not always suitable for EES, as EDLCs are non-Faradaic and capacitive.

## 3. Electrochemical Energy Storage (EES) Devices

Batteries and capacitors are two types of energy storage devices relevant to EES devices [29]. Essentially, batteries are non-rechargeable (primary cell) and rechargeable (RBs, secondary cell). On the other hand, capacitors are of three types—non-electrolytic, electrolytic, and electrochemical or supercapacitors (SCs). EES devices comprise RBs, SCs, and their derivatives. EES devices are different in that these devices store energy using different storage mechanisms—non-Faradaic (surface-controlled kinetics) and Faradaic (diffusion-controlled kinetics)—that depend on the materials (electrode and electrolyte) and how those materials are used in the device [8,21].

**Figure 7:**
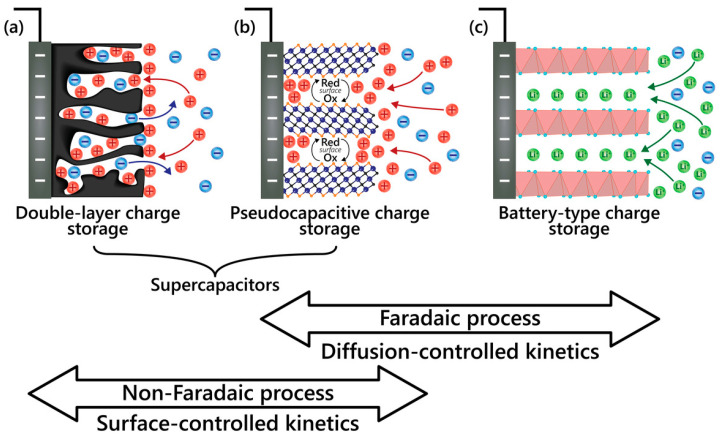
Illustration of the electrode processes occurring at (**a**) electrical double-layer capacitive, (**b**) pseudocapacitive, and (**c**) Faradaic electrodes [21].

Figure 7 illustrates three charge storage mechanisms and how those differentiate SCs from RBs. In a non-Faradaic process, charge accumulation occurs electrostatically with opposite charges residing on two interfaces separated by a vacuum (non-electrolytic) or a molecular dielectric (electrolytic). In contrast, a redox reaction achieves the same in a Faradaic process, causing chemical or oxidation state changes in the electroactive materials [3,29]. The Faradaic process can be capacitive (pseudocapacitive), as in PCs, or non-capacitive, as in batteries [19]. Before diving into supercapatteries, understanding three main EES—EDLCs, PCs, and RBs—is essential, as shown in Figure 7. The charge storage mechanisms in those devices can be well understood by their electrochemical signature in cyclic voltammetry (CV) and galvanostatic charge/discharge (GCD) profiles (Figure 3). Mathis et al. outlined a set of guidelines for interpreting the performance of EES systems [21]. An EDLC material will show a linear voltage versus time response (a triangular-shaped GCD profile) during constant current charging/discharging (Figure 3a) and a rectangular CV profile or cyclic voltammogram (Figure 3d). In this case, the amount of charge stored depends linearly on potential, and the capacitance of the material can be easily calculated and reported for the EDLC. On the other hand, an RB material will show plateaus in the GCD profile (Figure 3c) and separated oxidative and reductive peaks in the CV profile (Figure 3f). Unlike the case of charge storage at EDLC electrodes, charge storage by RB-type electrodes follows a nonlinear relationship with the applied potential. In the case of pseudocapacitive materials, the GCD profile (Figure 3b) is symmetric but non-linear, and the CV response (Figure 3e) does not separate the oxidative and reductive peaks.

### 3.1. Supercapacitors (SCs)

Depending on the storage mechanism, SCs can be classified mainly into three categories: EDLCs, PCs, and a combination of the two (HSCs [18,30,31,32,33] or asymmetric SCs (ASCs) [19,20,34], where HSCs are a subset of ASCs). Another way of differentiating ASCs from HSCs is that ASCs are configured by combining the electrode materials of EDLCs and PCs. In contrast, HSCs combine the electrode materials of EDLCs and RBs [35].

### 3.2. Electrical Double-Layer Capacitors (EDLCs)

**Figure 8:**
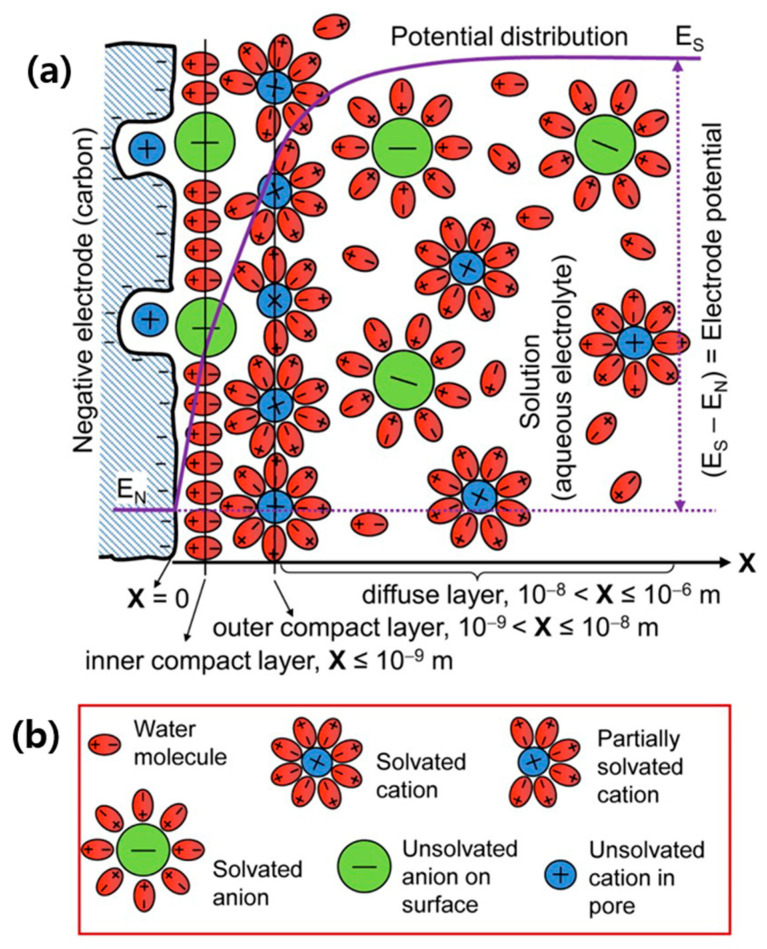
Schematic representations of (**a**) the EDL structure (cross-section) of the interface between a porous-carbon negative electrode and an aqueous electrolyte; (**b**) explanations of symbols in (**a**) [18].

Like conventional dielectric capacitors, EDLCs store energy electrostatically, forming an electrical double layer (EDL) at the electrode/electrolyte interface, where the applied voltage polarizes the electrolyte, which acts as a dielectric (Figure 8) [17,18,36,37,38]. The process is purely non-Faradaic and physical in nature. Thus, Equation (1) applies to EDLCs, and the charging–discharging mechanism of EDLCs is very fast and reversible. Additionally, EDLCs primarily utilize carbonaceous materials like activated carbon (AC), graphene, carbon nanotubes (CNTs), carbon aerogel (CA), carbide-derived carbon (CDC), carbon fibers, etc. [39] The capacitance of EDLCs mostly depends on the pore size of the electrode materials. Because of their porous electrodes with large surface areas that allow the formation of compact double layers with atomic range separation between electronic and ionic charges at the electrode surface, EDLCs show greater capacitance than conventional dielectric capacitors [36,39].

### 3.3. Pseudocapacitors (PCs)

**Figure 9:**
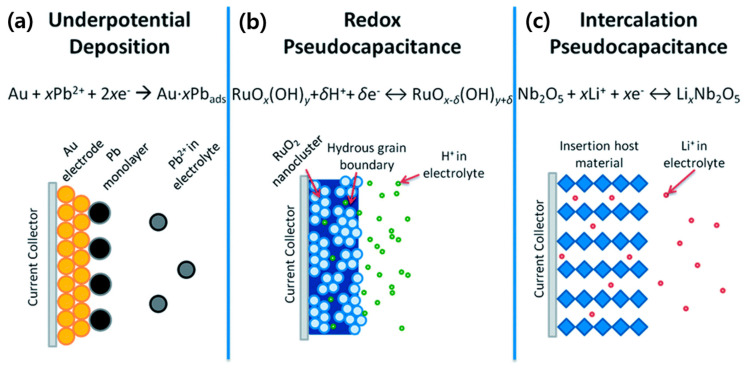
Different types of reversible redox mechanisms that cause pseudocapacitance: (**a**) underpotential deposition, (**b**) redox pseudocapacitance, and (**c**) intercalation pseudocapacitance [39].

In PCs, energy is stored via a sequence of fast reversible processes, which are Faradaic in nature, at the surface or near-surface of the electrode materials [8,40,41,42]. Conway identified three Faradaic mechanisms—underpotential deposition, electrosorption, and intercalation, that cause pseudocapacitance—as shown in Figure 9 [40,43]. In underpotential deposition, metal ions form an adsorbed monolayer at the surface of a different metal well above their redox potential. An example of such an underpotential deposition is the deposition of lead (Pb) on the surface of a gold (Au) electrode (Figure 9a) [44]. Redox pseudocapacitance occurs when ions are electrochemically adsorbed onto the surface or near the surface of a material following a Faradaic charge-transfer process (Figure 9b). Intercalation pseudocapacitance occurs when ions intercalate into the layers of a redox-active material in a Faradaic charge-transfer process without changing the crystallographic phase (Figure 9c) [40].

The above three Faradaic processes occur due to different physical processes involving various types of materials that result in similar electrochemical signatures owing to the relationship between potential and the extent of charge developed from adsorption/desorption processes at the electrode/electrolyte interface [40]:

(25) $$E\sim E^{0}-\frac{RT}{nF}\ln\left(\frac{X}{1-X}\right),$$

where *E* is the potential, *R* is the ideal gas constant, *T* is the temperature, *n* is the number of electrons, *F* is the Faraday constant, and *X* is the extent of fractional coverage of the surface or inner structure. From Equation (25), capacitance (*C*) may be defined in regions where the plot of *E* vs. *X* is linear:

(26) $$C=\frac{nF}{m}\frac{X}{E},$$

where *m* is the molecular weight of the active material. The capacitance, *C*, is not always constant, since the plot of *E* vs. *X* is not entirely linear as in a capacitor, and so is termed pseudocapacitance [40].

There are detailed reviews on PCs and related pseudocapacitive materials [39,41,42,45]. Electrode materials that are used in PCs include transition-metal oxides (TMOs) such as IrO_2_, RuO_2_, Fe_3_O_4_, MnO_2_, NiO, V_2_O_5_, Co_3_O_4_, etc.; transition-metal sulfides (TMSs) such as MoS_2_, WS_2_, and FeS_2_; and conducting polymers (CPs) such as polyaniline (PANI), polythiophene, polypyrrole (PPy), polyvinyl alcohol (PVA), poly (3,4-ethylene dioxythiophene) (PEDOT), polyacetylene, poly (4-styrene sulfonate) (PSS), poly-phenylene-vinylene (PPV), etc. [39,46]. Recently, many nanomaterials have been introduced to RBs that have shown fast redox kinetics comparable to pseudocapacitive materials due to the very short ionic diffusion length and high surface area of the nanosized materials. As a result, pseudocapacitive and battery materials are becoming increasingly indistinguishable [42]. According to Brousse et al., some materials are described as “pseudocapacitive” even though their electrochemical signature is analogous to that of a “battery material,” as commonly observed for Ni(OH)_2_ in KOH electrolytes. In contrast, true pseudocapacitive electrode materials such as MnO_2_ display electrochemical behavior typical of that observed for a capacitive carbon electrode [47]. Faradaic electrodes exhibit electrochemical behavior distinct from that of pseudocapacitive electrodes. Therefore, it is proposed that the term “pseudocapacitive” must be only used to describe electrode materials (e.g., MnO_2_) that display electrochemical behavior typical of that observed for a capacitive carbon electrode in a mild aqueous electrolyte to avoid any confusion between battery materials and pseudocapacitive materials.

### 3.4. Hybrid Supercapacitors (HSCs)

**Figure 10:**
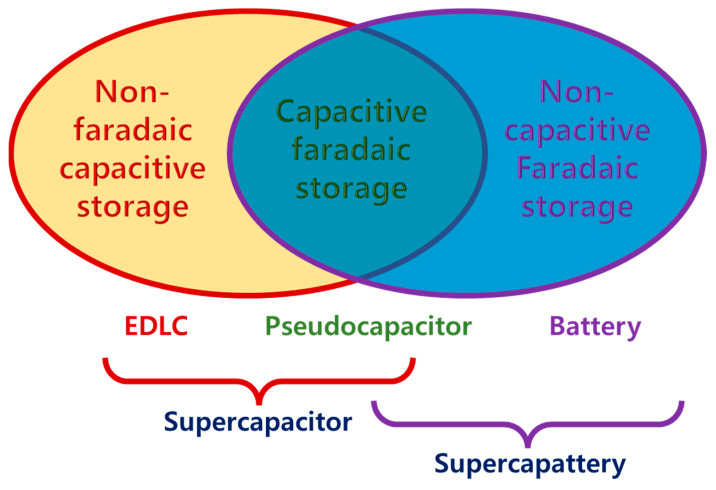
Schematic correlation between an EDL capacitor, pseudocapacitor, battery, and supercapattery according to charge storage processes [19].

Hybrid SCs (HSCs) take advantage of the best of EDLCs and the best of PCs or RBs in a single device with different combinations of electrode materials and storage mechanisms (non-Faradaic and Faradaic) (Figure 10) [19,20,48]. Hybridizing different electrode materials into a single electrode or fabricating a hybrid cell configuration consisting of Faradaic and non-Faradaic electrodes has become an obvious strategy for developing high-energy and high-power HSCs [8]. Thus, asymmetry in HSCs may arise from electrode materials and storage mechanisms. These devices take advantage of the fast kinetics of EDLC materials and the improved energy storage performance of pseudocapacitive or battery electrode materials [42]. Schematic illustrations of the electrochemical profiles (CV and GCD curves) of a capacitive asymmetric supercapacitor (different non-Faradaic materials) and a hybrid capacitor (non-Faradaic and Faradaic materials) are shown in Figure 4a,d, and Figure 4b,e, respectively.

### 3.5. Rechargeable Batteries (RBs)

Like most electrochemical devices, RBs are composed of two electrodes—a cathode and an anode—separated by an electrolyte [4,49,50,51]. In RBs, electrical energy is converted and stored electrochemically within the bulk of the electrodes through reversible chemical reactions at the electrode/electrolyte interface during the charging and discharging processes. There are various kinds of RBs; among them, lithium (Li)-ion batteries (LiBs), a type of metal-ion battery, are the most commercially successful RB technology [4,49].

**Figure 11:**
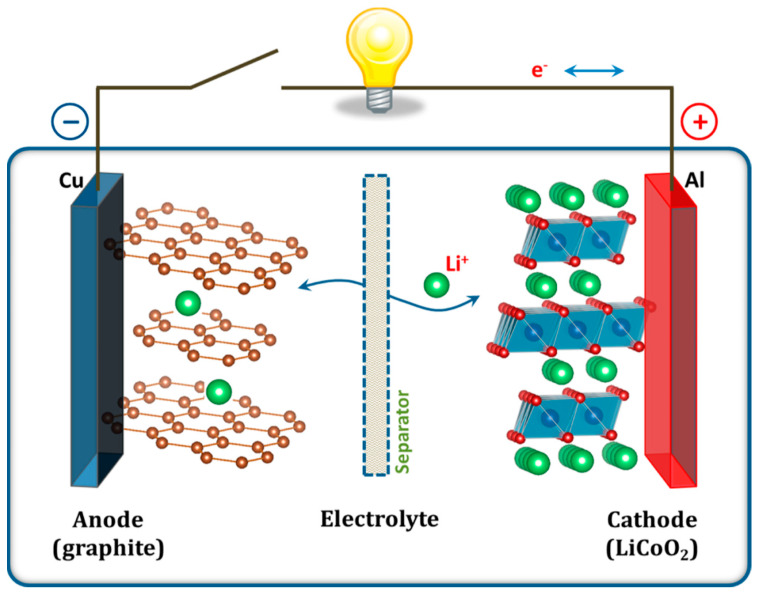
Schematic illustration of the first Li-ion battery (LiCoO_2_/Li^+^ electrolyte/graphite) [4].

**Figure 12:**
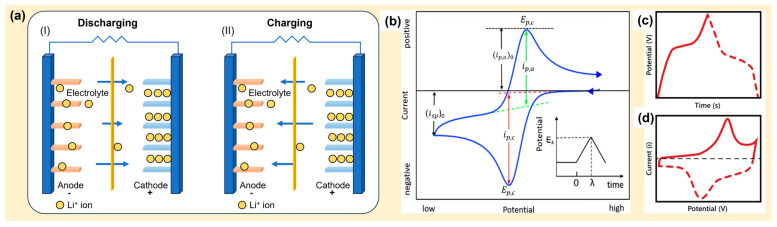
Schematic diagram of (**a**) the charge storage mechanisms ((I) charging and (II) discharging), (**b**) cyclic voltammogram (CV), and (**c**,**d**) galvanostatic charge-discharge (GCD) curves of LiBs [16].

The emergence of LiBs, a Nobel-Prize-winning technology and one of the most popular EES technologies in the nineties, has revolutionized consumer electronics and EVs [4,7,52,53]. Typically, LiBs comprise five key components—the anode, cathode, electrolyte, separator, and current collector—as shown in Figure 11 [4]. Generally, copper (Cu) and aluminum (Al) foils are used as current collectors at the anode and the cathode. The negative electrode (anode) is made of carbonaceous materials (e.g., graphite), whereas Li-based metal oxides (e.g., LiCoO_2_) are used in the positive electrode (cathode). Other materials used in the anode are germanium-based materials, transition metal chalcogenides, silicon, and metallic oxides [54]. The two electrodes are separated by a separator, typically a porous polyolefin film soaked in a non-aqueous solution of a lithium salt (e.g., LiPF_6_ in ethylene carbonate, ethyl methyl carbonate, or diethyl carbonate) [4,49]. In LiBs, the reversible chemical reaction occurs in two ways: displacement and insertion into the electrodes [4]. During the charging cycle, the positive electrolyte ions (Li^+^) are deintercalated (displaced) from the cathode and intercalated (inserted) into the anode. The reverse process occurs during the discharging cycle, where the positive ions transport from the anode to the cathode and electrons travel from the anode to the cathode via an external load and thereby complete the circuit (Figure 12a). This displacement/insertion process involves reversible Faradaic processes that can be identified as well-separated oxidative and reductive peaks in the CV profile (Figure 12b) and asymmetric curves in the GCD profile (Figure 12c,d). Emerging RBs based on abundant alkali, alkaline earth, and transition metals—sodium (Na), potassium (K), magnesium (Mg), calcium (Ca), zinc (Zn), and aluminum (Al)—are promising alternatives to LiBs [6,51]. Figure 4c,f illustrates the CV and GCD profiles of an RB.

### 3.6. Supercapatteries

Supercapatteries are hybrid EES devices that combine the advantages of SCs and RBs, such as high energy density, high power density, and a long cycle life. This EES hybrid design involves a combination of an SC electrode with an RB electrode, such as in the so-called Li-ion capacitor [55,56,57], Na-ion capacitors [24,56], and other hybrid EES devices [15]. Supercapatteries can exhibit capacitive performances like conventional capacitors, including CV and linear GCD profiles. Thus, the fundamentals of conventional capacitors can also be applied to supercapatteries, in which capacitance (C) is the ratio of the change in stored charge (ΔQ) to the variation in applied voltage (ΔV) as the voltage of a capacitor is swept at a constant voltage scan rate ($\nu =\frac{dV}{dt}$) in CV. Because the current (i) flowing through a capacitor is proportional to ν, this proportionality is also equal to *C*, as described in Equation (27) [15]:

(27) $$C=\frac{\Delta Q}{\Delta V}=\frac{dQ/dt}{dV/dt}$$

which is essentially the same as Equation (7). Figure 4b,e shows the schematics of the electrochemical profiles, including the CV and GCD curves of typical supercapatteries. Another term often used along with supercapattery is the “supercabattery”, which performs more like an RB but with higher power capability and/or longer charge–discharge durability [58,59,60,61]. Table 1 shows how different combinations of electrode materials with varying storage mechanisms give rise to supercapatteries [18]. Simon et al. pointed out that there should not be any confusion distinguishing EES devices—EDLSC, PCs, and RBs—since their electrochemical fingerprints are unique, as shown in Figure 3, Figure 4 and Figure 5 [62]. Supercapatteries are another class of EES combining the characteristics of both SCs and RBs.

### 3.7. Electrode and Electrolyte Materials in Supercapatteries

**Figure 13:**
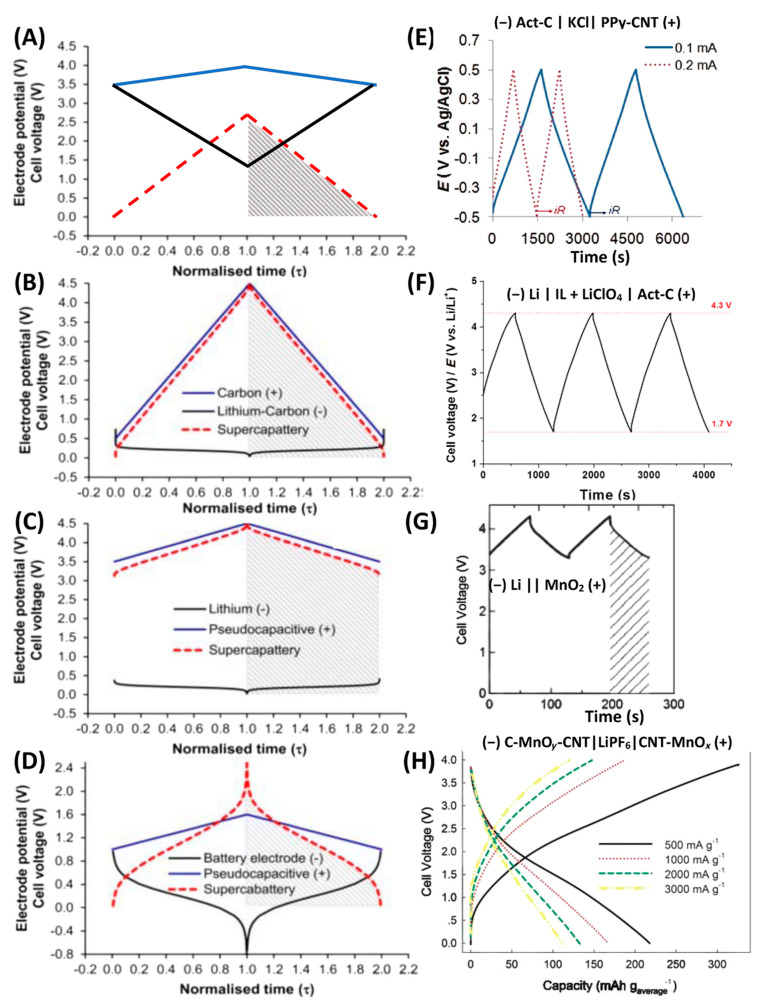
Calculated GCD plots for positrodes (blue lines), negatrodes (black lines), and relevant cells (red dashed lines): (**A**) a hypothetical pseudocapacitor with an Act-C negatrode and a pseudocapacitive positrode and a hypothetical supercapattery with a negatrode of Li metal or lithiated carbon and (**B**) an Act-C positrode or (**C**) a pseudocapacitive positrode. (**D**) A hypothetical supercapattery with a typical battery-type negatrode and a pseudocapacitive positrode. (**E**) Experimental demonstration of (**A**), (−) Act-C|KCl|PPy-CNT (+). (**F**) Experimental demonstration of (**B**), (−) Li|IL + LiClO_4_|Act-C (+). (**G**) Experimental demonstration of (**C**), (−) Li|PEO-LiTFSI|LTAP|1.0 M LiCl aq.|MnO_2_ (+). (**H**) Experimental demonstration of (**D**), (−) C-MnO_y_-CNT|LiPF_6_| MnOx-CNT (+) [15].

Different supercapatteries can be fabricated utilizing electrodes with capacitive, pseudocapacitive, or battery-type materials. Balasubramaniam et al. provided a comprehensive account of mechanisms, materials selection, and performance evaluation for supercapatteries [63]. Electrode materials with high surface area, electrical conductivity, porous structure, and short ion/electron diffusion lengths have essential characteristics for the performance improvement of supercapatteries. To comprehensively discuss these hybrid devices, Liu and Chen constructed several hypothetical supercapatteries to illustrate their performance using corresponding GCD plots, one by one (Figure 13A–D), and these hypothetical devices were confirmed using relevant experimental data from the literature (Figure 13E–H) [15]. Table 2 summarizes different supercapatteries based on their different electrodes and electrolytes, energies, and power densities. At the negatrode, mostly activated carbon of different sources was used in asymmetric battery/EDLC and pseudocapacitive/EDLC type supercapatteries. On the other hand, different metal iodides (BiOI-Bi_9_I_2_) [64], metal oxides (β-NiMoO_4_ [65], phosphate ion-functionalized NiO (P-NiO) [66], Zn_0.5_CoO_0.5_Mn(PO_4_)_2_ [67], Co_3_(PO_4_)_2_ [68], Co_0.5_Ni_0.5_WO_4_ [69], etc.), metal hydroxides (Co–Ni LDH [70]), metal sulfides (FeCoCuS_2_ [71], Co_0.125_Cu_0.375_Mn_0.500_S [72], etc.), composites (Co-MOF-PANI [73], Sr_3_P_2_-PANI [74], MWCNT-NiMnPO_4_ [75], graphitic carbon nitride (g-C_3_N_4_)-BiVO_4_ [76], Zn-Carbon cloths, [77], etc.), etc. were used for the positrode.

In the electrolyte, mostly, aqueous KOH at different concentrations has been used in supercapatteries [64,78,79,80,81]. Other electrolytes used include LiPF_6_ [77], K_2_SO_4_ [82], H_2_SO_4_ [83], KBr [82], NaClO_4_ [77], PVA-KOH [68], KI/VOSO_4_ [83], etc. Electrolytes with additional redox species have been investigated in supercapatteries with significantly enhanced energy capacity [31,58,84]. Also, EDLC materials with redox electrolytes showed enhanced performance [85].

**Table 2:** Performance of supercapatteries with different configurations. The data were collected from articles published during the last four years (2020–2023).

Device Configuration	Electrolyte	Electrode Type	Energy Density (Wh/kg)	Power Density (W/kg)	Publication Year	Reference
CeNiO_3_/Ni foam (symmetric)	6M KOH	(+)RB//RB(−)	38.70 28.41	774.81 7750	2022	[78]
CeNiO_3_/Ni foam (symmetric)	3M KOH	(+)RB//RB(−)	43.45	800	2022	[85]
BiOI-Bi_9_I_2_/Ni foam (symmetric)	6M KOH	(+)RB//RB(−)	38.2	2280.4	2020	[63]
β-NiMoO_4_/Ni foam (symmetric)	3M KOH	(+)RB//RB(−)	35.8 21.3	981.56 19,282.4	2020	[64]
Ni(py-TTF-py)(BPDC)/Ni foam//AC/NI foam	6M KOH	(+)RB//ED(−)	90.3 47.2	1180 10,400	2023	[86]
AgSr-Phosphate/Ni foam//CNT/Ni foam	1M KOH	(+)RB//ED(−)	55.02 42.37	741.54 9075	2023	[87]
Mxene/Ni foam//AC/Ni foam	1M KOH	(+)RB//ED(−)	68.8	1120	2023	[88]
NiS/Ni foam//AC/Ni foam	1M KOH	(+)RB//ED(−)	61.76	1275	2023	[89]
Fe_2_O_3_-α-Ni(OH)_2_/Ni foam//AC/Ni foam	1M KOH	(+)RB//ED(−)	44.51	2465	2023	[90]
CoMnS-rGO/Ni foam//AC/Ni foam	1M KOH	(+)RB//ED(−)	45.6	2880	2023	[91]
CoCuP/Ni foam//O, N, S-AC/Ni foam	6M KOH	(+)RB//ED(−)	37.3 18.4	915 12,308.8	2023	[79]
Fe-Mg MOF/Ni foam//AC/Ni foam	KOH	(+)RB//ED(−)	57	2393	2023	[92]
Ag_2_Co_3_(PO6)_2_/Ni foam//CNT/Ni foam	1M KOH	(+)RB//ED(−)	40.92 33.26	1237.5 4125	2022	[93]
SrS/Ni foam//AC/Ni foam	KOH	(+)RB//ED(−)	44.39 12.9	595 8400	2022	[94]
CeO_2_-ZnO-ZnWO_4_-AC/Ni foam//AC/Ni foam	2M KOH	(+)RB//ED(−)	56.92	2000	2022	[95]
MnS/Ni foam//AC/Ni foam	KOH	(+)RB//ED(−)	127.5	2550	2022	[96]
WS/Ni foam//AC/Ni foam	1M KOH	(+)RB//ED(−)	45.2	608	2022	[97]
NiCo LDH/Ni foam//AC/Ni foam	6M KOH	(+)RB//ED(−)	20.5 9.01	774.5 8522.7	2022	[69]
NiCo_2_S_4_-graphene/Ni foam//AC-graphene/Ni foam	4M KOH + carboxymethyl cellulose	(+)RB//ED(−)	80	4000	2022	[98]
LaMnO_3_/carbon cloth//rGO/carbon cloth	1M KOH	(+)RB//ED(−)	154 72	324 14,700	2022	[76]
LaMnO_3_/carbon cloth//rGO/carbon cloth	1M NaClO_4_	(+)RB//ED(−)	236	3630	2022	[76]
LaMnO_3_/carbon cloth//rGO/carbon cloth	2M LiPF_6_	(+)RB//ED(−)	123	1430	2022	[76]
NiFe-Phosphate/Ni foam//AC/Ni foam	1M KOH	(+)RB//ED(−)	45.6	2250	2022	[99]
2D MoO_3_-S_4_-C/Ni foam//rGO/Ni foam	6M KOH	(+)RB//ED(−)	129.6	11,600	2021	[77]
Cu-MOF-PANI-rGO-Ag/Ni foam//AC/Ni foam	1M KOH	(+)RB//ED(−)	52 27.2	1192 10,200	2021	[100]
Co_0.5_Mn_0.5_S/Ni foam//AC/Ni foam	1M KOH	(+)RB//ED(−)	61.34 9.92	850 8500	2021	[101]
VSB-5-rGO/Ni foam//AC/Ni foam	1M NaOH	(+)RB//ED(−)	80.5	2216.5	2021	[102]
FeCoCuS_2_/Ni foam//AC/Ni foam	3M KOH	(+)RB//ED(−)	48.2 27.2	820.1 25,700.2	2021	[70]
Co_0.125_Cu_0.375_Mn_0.500_S/Ni foam//AC/Ni foam	1M KOH	(+)RB//ED(−)	88.71 20	320 8000	2021	[71]
Co_3_(PO_4_)_2_/Cu/Ni foam//AC/Ni foam	1M KOH	(+)RB//ED(−)	62.6 11.8	425 7924	2021	[103]
NiMn(PO_4_)_2_-PANI/Ni foam//AC/Ni foam	1M KOH	(+)RB//ED(−)	71.3	340	2021	[104]
Co_3_(PO_4_)_2_/Ni foam//AC/Ni foam	1M KOH	(+)RB//ED(−)	34.8 10.0	425 6800	2021	[105]
MnCo_2_S_4_-Mxene/Ni foam//AC/Ni foam	3M KOH	(+)RB//ED(−)	25.6 12.44	400 6400	2021	[106]
Co_3_(PO_4_)_2_/Ag/Ni foam//AC/Ni foam	1M KOH	(+)RB//ED(−)	65.8	510	2021	[107]
Ni_0.75_Mn_0.25_(PO_4_)_2_/Ni foam//AC/Ni foam	1M KOH	(+)RB//ED(−)	64.2	340	2021	[108]
Co_0.125_Cu_0.375_Mn_0.500_(PO_4_)_2_/Ni foam//AC/Ni foam	1M KOH	(+)RB//ED(−)	56 16.88	800 6420	2021	[109]
Trypan blue-Ni-MOF/Ni foam//Azure A-graphene aerogel/Ni foam	3M KOH	(+)RB//ED(−)	66.55 11.11	349 4450	2021	[110]
NiO@CuCo_2_O_4_/MoNi/Ni foam//AC/Ni foam	2M KOH	(+)RB//ED(−)	80.6 63.8	692.8 14,000	2021	[111]
Zn_0.5_Co_0.5_S/Ni foam//AC/Ni foam	2M KOH	(+)RB//ED(−)	49 23	957 9413	2021	[112]
Co_0.5_Cu_0.5_Mn(PO_4_)_2_/Ni foam//AC/Ni foam	1M KOH	(+)RB//ED(−)	55 19	800 6400	2021	[113]
Ni-Co-Bi double hydroxide/Ni-Co-B/Ni foam//AC/Ni foam	2M KOH	(+)RB//ED(−)	62.8	800	2020	[114]
Phosphate ion-functionalized NiO (P-NiO)/Ni foam//AC/Ni foam	3M KOH	(+)RB//ED(−)	53.4 24.7	800 12,000	2020	[65]
Sr_3_P_2_-PANI/Ni foam//AC/Ni foam	1M KOH	(+)RB//ED(−)	28.9 10.95	1020 5100	2020	[73]
Zn_0.5_CoO_0.5_Mn(PO_4_)_2_/Ni foam//AC/Ni foam	1M KOH	(+)RB//ED(−)	45.45 6.86	425 4250	2020	[66]
Co_3_(PO_4_)_2_/Ni-Co-O/Ni foam//Fe_2_P/graphene hydrogel/Ni foam	PVA-KOH	(+)RB//ED(−)	95 18	400 4000	2020	[67]
Co_0.5_Ni_0.5_WO_4_/Ni foam//AC/Ni foam	2M KOH	(+)RB//ED(−)	42.2	1047.7	2020	[68]
Co-MOF-PANI/Ni foam//AC/Ni foam	1M KOH	(+)RB//ED(−)	23.11 8.906	1600 6400	2020	[72]
WO_3_-WS_2_-MWCNT/Ni foam//AC/Ni foam	3M KOH	(+)PC//ED(−)	86 24	848 11,828	2023	[115]
Ni-Co-Mg MOF/MoS_2_/Ni foam//AC/Ni foam	1M KOH	(+)PC//ED(−)	107.32	1350	2023	[116]
NH_4_MnPO_4_@Graphene QD/Graphite//rGO/Graphite	3M H_2_SO_4_ 3M H_2_SO_4_ + 0.025M (KI/VOSO_4_)	(+)PC//ED(−)	199 311	450 450	2022	[82]
Ni_3_(PO_4_)_2_-MWCNTs/Ni foam//AC/Ni foam		(+)PC//ED(−)	94.4 24.82	340 10,200	2022	[117]
Mn-V-Sn oxyhydroxide/Ni foam//N-carbon/Ni foam	1M KOH	(+)PC//ED(−)	70.6 17.1	1372.4 18,861.3	2022	[118]
NH_4_OH-ZIF/Ni foam//GO/Ni foam	6M KOH	(+)PC//ED(−)	4.16	20,000	2022	[119]
CoS-Co_3_(PO_4_)_2_/Ni foam//AC/Ni foam	1M KOH	(+)PC//ED(−)	34.68 63.93	13,600 850	2021	[120]
Fe_3_O_4_@N-carbon-rGO/Ni foam//rGO/Ni foam	6M KOH	(+)PC//ED(−)	46 10	750 7500	2021	[121]
MWCNT-NiMnPO_4_/Ni foam//AC/Ni foam	2M KOH	(+)PC//ED(−)	698 43	78 5780	2020	[74]
P-NiCoB/Ni foam//rGO/Ni foam	2M KOH	(+)RB//PC(−)	63.125 41.56	750 15,000	2023	[80]
CoMn_2_O_4_/N-graphene/Ni foam//N-graphene/Ni foam	PVA-KOH	(+)RB//PC(−)	44.1 20.3	992.6 12,430	2021	[122]
graphitic carbon nitride (g-C_3_N_4_)-BiVO_4_/Graphite paper (symmetric)	3.5M KOH	(+)PC//PC(−)	61 7.2	1996 16,200	2020	[75]
Zn-Carbon cloths//S/P doped carbon (S/p-C)/graphite rod	0.5M K_2_SO_4_ 1M KBr	(+)PC//PC(−)	270 181	185 9300	2020	[81]

ED → electrical double-layer capacitive type. PC → pseudocapacitive type. RB → rechargeable battery type.

### 3.8. Performance and Experimental Evaluation of Supercapatteries

**Figure 14:**
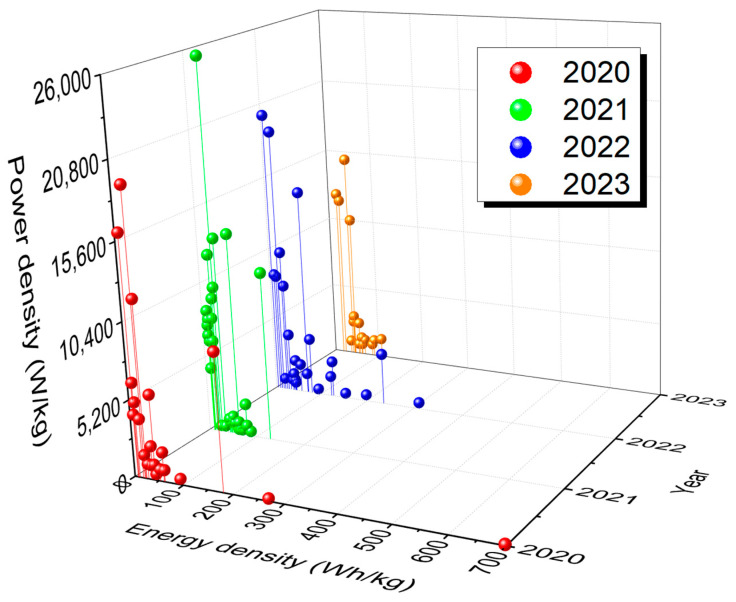
Ragone plots for different supercapatteries reported over the last four years (2020–2023) showing relative energy and power density.

Figure 14 shows Ragone plots of supercapatteries reported in published articles during the last four years, from 2020 to 2023. The corresponding data are summarized in Table 2. Primarily, the reported supercapatteries performed either like an EDLC or an RB. However, Devi et al. reported a Na-ion supercapattery with outstanding specific energy of 236 W h kg^−1^ at a higher specific power of 3630 W kg^−1^ with appreciable retention of over 95% even at the 10,000th cycle [77]. It is important to note that capacitance can be used only when there is a linear relationship between charge and voltage, and the capacitance value should be a single constant value in the chosen potential window; any deviation from this behavior requires that integration be used to calculate the charge being stored or delivered. Also, capacity instead of capacitance should be measured for RBs. As Chen has pointed out, many authors have ignored the critical differences between SCs and RBs as they have applied the concept of pseudocapacitance to some new battery-type materials [18]. As a result, deceptively high specific capacitance values have been claimed, and high specific capacitance has been also used in calculating specific energy.

### 3.9. Classification of EES Devices

**Figure 15:**
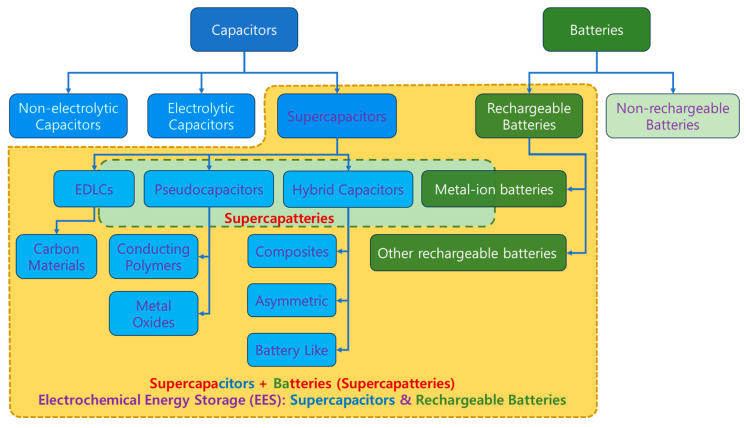
Various electrochemical energy storage (EES) systems based on storage mechanisms [8]. EES and supercapatteries are highlighted in yellow and green rectangles bordered with dashed lines [47].

From the above discussion, it is evident that RBs and SCs belong to EES, as shown in Figure 15, where EDLCs and PCs belong to SCs. However, placing HSCs and supercapatteries in the classification tree is inconsistent throughout the literature [8,12,18,20,30,31,32]. Even ASCs have been placed parallel to HSCs or in place of HSCs, making HSCs a subclass of ASCs [20]. Initially, HSCs appeared to be defined as hybrids of EDLCs and PCs, whereas supercapatteries were considered hybrids of EDLCs and RBs. Guan et al. proposed defining and differentiating storage mechanisms in EES devices according to Figure 10 without explicitly differentiating ASCs and HSCs [19]. Considering all possible combinations of electrode materials and storage mechanisms, EES devices were classified vividly in tabulated form (Table 1) [18], where the combination results in nine different devices depending on electrode materials and storage mechanisms.

## 4. Summary and Perspective

All supercapatteries are hybrid EES devices, but not all hybrid EES devices are supercapatteries. Supercapatteries are envisioned as a technology that will mutually complement the drawbacks of RBs and SCs. Supercapatteries are EES devices that can integrate the benefits of RBs and SCs using all three charge storage mechanisms: non-Faradaic capacitive storage (EDL capacitive storage), capacitive Faradaic storage (pseudocapacitive storage), and non-capacitive Faradaic storage (rechargeable battery-type storage or Nernstian charge storage). Moreover, supercapatteries can be made of non-Faradaic capacitive EDLCs using a redox electrolyte. However, it is essential to develop new methods and materials to minimize the processing and manufacturing cost of these devices. Future research should concentrate on the fabrication of supercapatteries by selecting nanostructured carbon materials, metal oxides, and electrolytes with excellent electrochemical performance. In summary, supercapatteries have gained increasing attention in the EES field, as seen in the growing number of publications using the term “supercapattery.” The development of supercapattery materials can benefit from advances in battery and supercapacitor materials. Nanotechnologies and engineering will play a more significant role in advancing micro-supercapatteries in powering IoT devices. Like LiBs, supercapatteries are on the verge of revolutionizing the whole ecosystem of energy storage and related industries, including renewable energy, portable electronics, and the EV industry.

## Figures and Tables

**Table 1:** Ways of pairing the same or different electrode materials into a supercapacitor, battery, supercapattery, or supercabattery [18].

**Device**			**Supercapattery**						Battery
	**Supercapacitor**					**Hybrid**		Supercabattery	
	**EDLC**		**Pseudocapacitor**						
**Electrode Material**	**NFCS**	**NFCS**	**NFCS**	**CFS**	**CFS**	**NFCS**	CFS	NCFS	NCFS
	1 + 1	1 + 2	1 + 3	1 + 1	1 + 2	1 + 3	1 + 1	1 + 1	1 + 2
	NFCS	NFCS	CFS	CFS	CFS	NCFS	NCFS	NCFS	NCFS

**NFCS:** Non-Faradaic capacitive storage (EDLC storage). **CFS:** Capacitive Faradaic storage (pseudocapacitive storage); **NCFS:** Non-capacitive Faradaic storage (battery-type storage); **1 + 1:** Symmetrical device of the same electrode material; **1 + 2:** Asymmetric device using different materials with the same storage mechanism; **1 + 3:** Asymmetrical device using different materials and different storage mechanisms.
